# Mental health among farmers in Germany: a scoping review

**DOI:** 10.3389/fpubh.2025.1701468

**Published:** 2025-12-17

**Authors:** Meike Heming, Louisa Scheepers, Kira Schmidt-Stiedenroth, Peter Angerer

**Affiliations:** Institute of Occupational, Social and Environmental Medicine, Centre for Health and Society, Medical Faculty and University Hospital, Heinrich-Heine-University Düsseldorf, Düsseldorf, Germany

**Keywords:** agriculture, farmers, mental health, depression, stress, burnout, Germany

## Abstract

**Background:**

Farmers are exposed to various stressors, including economic uncertainty, climate variability, and regulatory burdens, all of which can negatively impact their mental health. While international research has documented elevated rates of mental illness among farmers, little is known about the mental health status of farmers in Germany. This scoping review aims to synthesize current scientific knowledge on mental health outcomes among farmers in Germany.

**Methods:**

A scoping review was conducted according to PRISMA guidelines for Scoping Reviews and following the Population Concept Context (PCC) framework. Inclusion criteria were: (a) language: English or German, (b) publication between 2014 and 2024, (c) original study, (d) population: farmers (at least 50% within the study population), (e) concept: mental health and (f) context: Germany. Web of Science and Google Scholar were chosen as databases, complemented by expert input and reference checking of relevant studies.

**Results:**

Eleven studies met the inclusion criteria: six cross-sectional studies, three randomized controlled trials, and two qualitative studies. The outcomes reported were: depressive symptoms, anxiety and burnout symptoms, sleep disorders and perceived stress. Identified stressors linked to the outcomes included economic difficulties, bureaucratic pressure, climate-related challenges, and family conflict. However, most studies showed methodological limitations and heterogeneity regarding definitions and instruments. No data were found on suicide among German farmers.

**Discussion:**

Compared to international research, evidence on mental health of farmers in Germany remains limited. The few mental health findings among German farmers align with those from international studies. Further epidemiological studies and context-specific interventions are needed to address mental health of farmers in Germany.

**Systematic review registration:**

https://osf.io/yagbp/?view_only=

## Introduction

Agriculture is an occupational sector with a number of unique working conditions and challenges. The most common health risk factors for farmers in developed countries described in the literature include pesticide exposure, financial problems, bureaucratic pressure, unpredictable climate (extreme weather conditions, e.g., droughts), injuries, poor physical condition, animal diseases, machinery failure, time pressure, long working hours and government regulations and inspections (1, 2). In particular, the use of pesticides, financial problems, climate change and physical condition or injuries are associated with poor outcomes for farmers’ mental health or mental illness (2–4). Generational conflicts have also been identified as stressors, with evidence suggesting that the stress experienced by parents frequently extends to their adolescents (5).

Agriculture is therefore regarded as a particularly stressful profession (6), and for several years now, there have been global concerns about the rising rates of mental health problems in this occupational sector (1). A number of global studies - predominantly from developed countries in North America, Australia and England - show that farmers have poorer mental health than the general population (1–5). Prevalence rates are particularly high for mental disorders such as anxiety and depression (5). Poor mental health among farmers is not only stressful for the individuals, but also has an impact on their families, who often find it difficult to cope with the symptoms. It also affects the productivity and ultimately the existence of the farm business and the welfare of the animals, which may be neglected as a consequence (1, 3). The results of an American cross-sectional study, for example, showed a strong correlation between depressive symptoms of farming parents and depressive symptoms of their adolescent children (5). Due to the fact that also the children live on the farm, they are ultimately often already exposed to the same health risk factors as their parents. Further, they often support the work of their parents and are constantly confronted with the farm’s problems (financial, government regulations) (5). The same conditions may apply for the older generation, i.e., the parents of the farming parents, which can also contribute to work- and private-related conflicts.

When it comes to recording and reporting prevalences, difficulties arise as different distinctions are made between main occupation farmers and part-time farmers. Further, the associated data is recorded by different authorities. In addition, different terminology at international and national level makes it difficult to precisely describe and identify prevalences. Internationally, the term farming is often used to summarize all areas of agriculture (e.g., crop farming, forestry, fishing, all animal farming, organic and conventional farming, green sector) (7).

Agriculture is a male-dominated sector (20–40% women worldwide) (8). Due to the fact that men often have the leading position on farms and own the farmland (8), they are faced with considerable work-related stress due to the complex interaction of social, environmental and economic factors (2, 6). Occupational stress and the resulting strain can arise when work demands are high and are not accompanied by an appropriate level of control to cope with these demands (9). Control and self-efficacy, including control over their own health, are important to male farmers, which is why they prefer to manage mental disorders themselves and only reluctantly accept outside help (6). However, this often leads to attempts to cope with depressive symptoms through social isolation and substance use (6). In addition, suicidal behaviour is also described as a way of coping or giving up to cope with symptoms of mental disorders (4, 6). Studies around the world show that farmers commit suicide more frequently (also when considering work-related stress) than the general population (2, 4). For example, the suicide rate among French farmers is at least 20% higher than in the general population (10) and in Switzerland the suicide risk among farmers is 37% higher than among other men from a rural community (11). One possible explanation for the high suicide rates among farmers discussed in the literature is the easy access to toxic substances and weapons in the broader sense (e.g., machinery) (4).

With around a third of the world’s population working in agriculture (12), poor mental health among this group can have a hugely negative impact on society’s economic productivity, food security and therefore global health. Therefore, it is of utmost importance that evidence on the risks for developing (mental) health related disorders is investigated internationally and nationally. By doing so, it will be possible to protect farmers health and safety at work as well as to improve it, if necessary. Ultimately, the health of farmers will affect not only the individual farmers and their families but also the societies, which benefit from their food production. For example, Germany is one of the biggest agricultural producing country in the European Union (13, 14) and around 1.2% of the population in Germany worked in agriculture in 2023 (15). Due to modern agricultural possibilities, these farmers cultivate half of Germany’s total area (16). As a result of structural change in agriculture, the number of farms with large farmland is increasing and smaller farms are disappearing, so that today one farmer feeds 147 individuals in Germany (17). However, it seems that evidence on German farmers is absent from international reviews investigating their mental health (1–4). Due to different occupational definitions worldwide regarding who belongs to the agricultural sector, as well as different laws, working conditions and health systems, it seems necessary and helpful to gather information and provide an overview on evidence on farmers mental health in Germany.

For example, the German Classification of Occupations 2010 classifies the occupational groups for agriculture, forestry, animal husbandry and horticulture separately (18). However, information on farmers is often summarized into the so-called “*green professions”* (19). For our work at hand, farming entails: agriculture that involves crop and livestock (dairy, egg, and meat producing) farming, excluding garden and landscaping contractors, gardeners, equine professionals and forestry workers to focus on food production to be able to depict potential similar working conditions (15). The above mentioned international evidence on farmers mental health and the associated risk factors for the farmers themselves and society at large make clear that obtaining such evidence for the German context is of utmost relevance from a Public Health perspective. Such knowledge is required to take targeted measures to prevent mental health problems and provide assistance. Given that unpredictable natural events will increase due to climate change, the risks to farmers’ mental health are expected to increase further (4, 20). In regard of this fact, it is striking that no studies from Germany are considered or mentioned in international reviews (see: 1–4). In addition, it seems that there is a lack of precise values on the prevalence of mental disorders among farmers in Germany.

Thus, to systematically identify and provide an overview of the scientific findings to date on mental health among farmers in Germany and to highlight the resulting research implications we conducted a scoping review. To achieve these aims, the following research questions were addressed:

(1) Which mental health outcomes are reported among farmers in Germany, and how prevalent are they?
(2) What potential research implications result from the findings?

Due to the possible methodological simplification, scoping reviews represent a practicable and resource-efficient approach to gain an overview of the current literature and evidence base for a topic which seems to show relevant research gaps (21, 22). For conceptualization, the Population Concept Context (PCC) framework (Population: farmers, Concept of interest: mental health, Context: Germany) was used (23).

## Methods

To conduct this scoping review, we followed recommendations from Peters et al. (23) according to the guidelines for conducting systematic scoping reviews and have based our review mainly on the PRISMA Checklist for scoping reviews (PRISMA-ScR) (24) and will clarify within the method section where our approach has differed (24, 25). The PRISMA-ScR Checklist is provided in the Supplementary material, Data Sheet 1. Following recommendations, we developed a study protocol prior to conducting this review and registered it in the Open Science Framework (https://osf.io/yagbp/?view_only=) (23). The protocol is provided in the Supplementary material, Data Sheet 2. During the review process, amendments were made to the protocol (see Supplementary material, Data Sheet 3); details and rationale for these changes are reported in the section ‘Protocol amendments’.

### Eligibility criteria

To be included in the scoping review, articles had to be published either in English or in German, the articles had to be published within 2014 and 2024 and had to be original studies. The time frame was chosen on the basis of previously published reviews (2, 3, 26), which show that there was a significant increase in publications on the topic of mental health among farmers, particularly around 2014, and that since then the number of publications has remained at a consistently high level or has increased. The period was also chosen in order to capture the most recent evidence and reflect the current state of mental health among farmers. Further, farmers had to be included as the study population (comprising at least 50%) via data collection and the concept of mental health had to be investigated/considered. Thus, also green professions could be included in the scoping review, if farmers comprised at least 50% of the study population. Green professions can account for farmers, garden and landscaping contractors, gardeners, equine professionals, forestry worker, livestock farmers or agricultural service technicians, for example (27). In addition, we included women working or living on farms, as they are known to be involved in all farm activities and contribute significantly to the economic success of the farm (28, 29). We thereby decided on broad terms for the search strings to be able to include a large number of potential studies (details under search strategy). Thus, we were interested in outcomes such as depression, stress, anxiety, burnout or suicide. At last, main data collection had to be conducted in Germany to ensure that the concepts assessed are relevant for the context of farmers in Germany.

We have accordingly applied the following exclusion criteria: Other language than English or German, publication outside of the specified time period, article is not an original study, farmers are not included as the study population, the concept mental health is not investigated and/or data was not collected in Germany.

### Information sources

We have chosen Web of Science (last search September, 20th 2024) and Google Scholar (last search September, 20th 2024). Web of Science was used because it provides comprehensive, high-quality coverage of peer-reviewed studies across social sciences, health, and environmental disciplines, making it well-suited to identify research especially for mental health among particular occupational groups. Google Scholar was chosen to further identify German sources and because it is recommended in the literature for identifying grey literature, in particular (30). For additional sources, experts were consulted one time on September, 20th 2024 to retrieve information for additional studies or grey literature. These experts worked at the German Social Insurance for Agriculture, Forestry and Horticulture and provided additional potential articles. Further, for all screened full-texts, we conducted reference checking to identify additional studies that may have been missed by our search strategy.

### Search strategy

The search string was developed according to the PCC framework: farmers were chosen as the population, mental health was the chosen concept and the context was Germany. To not miss any potentially relevant articles, we tried to create sensitive search strings which would cover a broad range regarding our concept of mental health.

In Web of Science, the following search strategy was applied for topic search (TS; including title, abstract, author keywords and keywords plus): ((TS = (farm*)) OR TS = (agriculture)) AND (((((TS = (mental health)) OR TS = (depress*)) OR TS = (suicid*)) OR TS = (burnout)) OR TS = (stress)) OR TS = (anxiety) AND TS = (german*). This search was filtered for the publication date being within 2014-09-20 and 2024-09-20 and language English OR German.

In Google Scholar, the search was conducted in German only and restricted toward the first 200 references (31). The search strategy was the following: farmer AND mental health AND Germany. This search was filtered for the publication year being within 2014 and 2024 and the language was set to German. The review protocol (available on https://osf.io/fc8aq) illustrates the search strategy and filters used in Google Scholar in German language. By conducting the search in Google Scholar, it was aimed to also receive reports from German organizations, insurances or universities (e.g., theses). Google Scholar was included with the specific aim of capturing grey literature which could behighly relevant to the German agricultural context but may not be indexed in traditional academic databases. As preliminary searches indicated that these types of publications were mainly written in German, we believe this language choice therefore increased the precision and contextual appropriateness of the Google Scholar results.

### Selection process

Retrieved records were exported from the two databases into an online management tool for systematic reviews (rayyan.ai). The software automatically detected duplicates, which were then manually checked by MH, KSS and LS and removed from the management tool. To pilot the title and abstract screening process, 50 records were screened in parallel by KSS and LS (21, 22), disagreements were discussed with MH. The remaining 381 records (title and abstract) were screened by LS. In case of doubt, LS consulted MH and KSS to discuss and decide upon inclusion or exclusion. The 24 records selected for the full-text screening were all screened by LS, MH and KSS. In case of discrepancies, the full-texts were discussed in a team of two reviewers, and a third reviewer was consulted, when two reviewers did not come to an agreement until consensus was reached. If more than one exclusion criteria applied, only one criterion was documented in the management tool according to the following order: wrong language, wrong study period, no original study, wrong population, wrong concept and data collection not in Germany. After full-text screening, reference checking was done individually by the three reviewers to find additional records.

### Data collection process and data extraction

In order to collect data from the retrieved records, a data extraction sheet was developed by KSS based on recommendations for a scoping review (23). The sheet documented key information of the studies relevant to the research aim (23). Reviewers were colour-coded and the following information was documented by all three reviewers independently: Date when reviewed, authors, year of publication, country origin, aims of the study, defining characteristics of the study participants and sample size (if applicable), type of study, specific methodology, setting and context related information, concepts used to measure mental health (if applicable), measurement of outcome, key findings, author’s conclusion and at last reviewer’s comments. After documentation of all relevant findings, MH and LS discussed and compared the key information, checked accuracy and excluded further studies, if during the review process exclusion criteria became evident. If LS and MH came to a disagreement, KSS was consulted and cases were discussed together until agreement. The data sheet built the foundation for the following working steps and supported the development of a further table for description of assessed measurements.

### Synthesis methods

For quantitative studies we report assessed outcomes as presented in the studies, focusing on mean values and standard deviations, or prevalence, depending on which data the authors of the studies reported. Otherwise, we report results of quantitative, qualitative or mixed-methods studies narratively. We mention which mental health outcomes are reported, including related concepts such as potential stressors causing the mental health burden (if reported), to synthesize the most relevant information. The categorization of results was derived from the analysed data and findings. To facilitate readability, we show results in a table format summarising the assessed outcomes from the more detailed data extraction sheet into overall topics (Table 1). Results are also presented narratively according to the study design. Due to the heterogeneity of studies and the reported data, a common classical quantitative synthesis was not possible and was not the aim of this scoping review.

**Table 1:** Overview of the included studies (2014–2024).

Reference nr.	Author(s)	Publication year	Language	Type of publication (Study design)	Study population (*n*)	Recruitment process/inclusion criteria	Stressors mentioned alongside mental health	Mental health outcomes
(32)	Roth	2021	German	Published article based on master’s thesis (cross-sectional stuagriculturaldy)	Farmers (*n* = 2,788)	Recruitment: by flyer (via social media and personal contacts) and via newspaper “Bayerisches Landwirtschaftliches Wochenblatt “(a Bavarian farmers’ newspaper). Inclusion criteria: not reported.	Not reported	Depression Anxiety Burnout
(33)	Betz, Engemann and Vu	2022	German	Original published article (cross-sectional study)	Trainees of green professions (*n* = 2.662)	Not reported	Bureaucratic and regulatory stress factors (time pressure) Social and family challenges (conflicts with colleagues or superiors, work climate) Working conditions (heavy lifting, work climate)	Depression Sleep disorders General health status
(34)	Knoop and Theuvsen	2020	German	Original published article (cross-sectional study)	managers and owners of agricultural and horticultural enterprises (*n* = 288)	Recruitment: by survey link that was spread via farmer’s newspaper, social media, e-mail lists of farmers (working groups and agricultural chambers and offices (convenience sample) Inclusion criteria: not reported.	Bureaucratic and regulatory stress factors Societal challenges (social environment) Working conditions (work intensity, physical effort)	Not reported
(35)	Braun et al.	2021	English	Original published article (randomized controlled trial)	Green professions (*n* = 360)	Recruitment: pragmatic (simultaneously to Braun et al. (36), see below) Recruited almost nationwide (not in Bavaria and Schleswig-Holstein); January 2018 to April 2019 Policyholders were invited by postal letter and e-mail (N = 80.000) send by insurance, or self-selection recruitment channels via farmers´ articles, newsletter postings, online-information or several websites Inclusion criteria: Policyholders from SVLFG (German Social Insurance for Agriculture, Forestry and Horticulture) Perceived Health Questionnaire (PHQ-9) ≥ 5 ≥ 18 years No psychotherapy treatment Working/ Employed, family member or pensioner with adequate insurance status Web access available Ability to step back from suicidal ideation Preferred this trial over parallel chronic pain study (if applicable)	Not reported	Depressive symptoms Anxiety Burnout/ Emotional exhaustion Life satisfaction Sleep disorders General health status (mental health care service use) Perceived stress
(36)	Braun et al.	2022	English	Original published article (randomized controlled trial)	Green professions (*n* = 81)	Recruitment: pragmatic (simultaneously to Braun et al. (35), see above). Recruited almost nationwide (not in Bavaria and Schleswig-Holstein); starting in February 2018 Policyholders were invited by postal letter and e-mail (N = 80.000) send by insurance, or self-selection recruitment channels via farmers´ articles, newsletter postings, online-information or several websites. Inclusion criteria: Policyholders from SVLFG (German Social Insurance for Agriculture, Forestry and Horticulture) Pain duration at least 6 months Considerable pain intensity: at least grade II according to the Chronic Pain Grade questionnaire (CPG) ≥ 18 years E-mail address available Web access available Willing to consent	–	Depressive symptoms Anxiety Life satisfaction Sleep disorders General health status (mental health care service use) Perceived stress
(37)	Schöllhorn	2020	German	Published article based on bachelor’s thesis (cross-sectional study)	Green professions (*n* = 634)	Recruitment: not reported Inclusion criteria: not reported	Environmental and climate-related stressors Bureaucratic and regulatory stress factors (mental and time-related) Social and family challenges Personal stressors (for male: high requirements and restrictions; for female: family conflicts, peaks in workload, liquidity problems) Working conditions (lack of sleep, working with animals)	Perceived stress
(38)	Stier-Jarmer et al.	2020	English	Original published article (randomized controlled trial)	Green professions (*n* = 108)	Recruitment: convenience sample Inclusion criteria: Increased stress and increased risk for physical and/or mental health impairments (assessed by physician) SVLFG (German Social Insurance for Agriculture, Forestry and Horticulture)-membership ≥ 18 years Working/ Employed Physically and mentally stable, moderately resilient	Not reported	Depression/ Well-being Anxiety Perceived stress Burnout symptoms Sleep disorders Health status
(39)	Hetzel et al.	2016	German	Original published article (cross-sectional study)	Green professions (*n* = 3.176)	Recruitment: a three-stage random sample, stratified by regional population, was taken from the SVLFG (German Social Insurance for Agriculture, Forestry and Horticulture) address database Inclusion criteria: ≥ 55 years Employed or working in the Bavarian agriculture	Social and family challenges (generational conflicts)	Life satisfaction General health status (psychological complaints)
(40)	Buhne	2019	German	Published article based on master’s thesis (qualitative interview study)	Farmers (*n* = 6), wife of a farmer who committed suicide (*n* = 1, treated as farmer in analyses)	Recruitment: not reported Inclusion criteria: Burnout treatment received	Economic stressors (pressure, competition among farmers) Bureaucratic and regulatory stress factors Societal challenges (poor social support among colleagues, social and political pressure) Social and family challenges (work-life conflicts, conflicts regarding farm transfer) Personal stressors (rejection of mental health problems of older generations)	Burnout Sleep disorders General health status (physical symptoms, performance capability)
(41)	Greiner	2024	German	Published article based on master thesis (qualitative interview study)	Individuals from the agricultural sector (*n* = 4) and consultants (*n* = 4)	Not reported	Economic stressors Bureaucratic and regulatory stress factors Social and family challenges (poor communication, intergenerational conflicts, role conflicts, conflicts between couples, missing or unwilling heirs) Personal stressors (role transition)	Not reported
(42)	Von Davier	2024	German	Original published article (cross-sectional study)	Women who live and/or work on farms (*n* = 5.488)	Recruitment: not reported, nationwide survey Inclusion criteria: Women living and/or working on farms Women who have a connection to the farm either through: family member, existing employment or apprenticeship	Economic stressors Societal challenges Social and family challenges Work conditions	Burnout

We tried to consider and limit potential reporting bias by including studies in German and English and grey literature by conducting the search in Google Scholar and by consulting experts in the field. However, as the purpose of this scoping review was to provide an overview of the existing literature on the topic, we have not used other measures to assess potential reporting bias. As it was aimed to achieve a first overview of mental health among famers, it was decided to not perform a risk of bias assessment. We report all relevant study characteristics regarding design, context and population sample, if this information is mentioned in the assessed studies and otherwise report when this is not the case.

### Protocol amendments

The review protocol was amended after the initial literature exploration (Supplementary material, Data Sheet 3). Originally planned as a rapid review, the methodology was adapted to a scoping review. The literature on mental health among farmers in Germany turned out to be broader and more heterogeneous than anticipated, with considerable variability in study designs, populations, and quality. This heterogeneity limited the feasibility of synthesizing findings within a rapid review framework. Adopting a scoping review approach allowed us to comprehensively map the available evidence and identify research gaps, ensuring that the review remained methodologically appropriate and maximally relevant for future research and practice.

## Results

### Included studies

**Figure 1:**
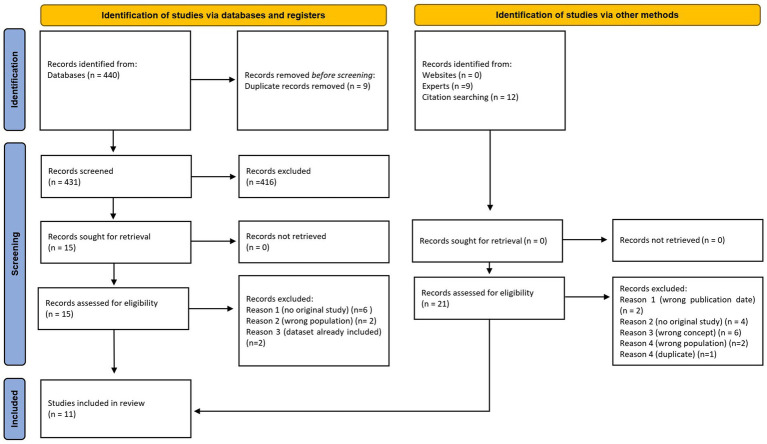
Flowchart of excluded and included studies. Source: Page MJ, et al. BMJ 2021;372:n71. doi: 10.1136/bmj.n71. This work is licensed under CC BY 4.0. To view a copy of this license, visit https://creativecommons.org/licenses/by/4.0/.

The search conducted in September 2024 yielded a total of 440 records/hits: 240 results were found in Web of Science and from the search in Google Scholar we used only the first 200 hits, as recommended (30, 31). Additional consultation with experts of the German Social Insurance for Agriculture, Forestry and Horticulture yielded a further nine records. The title and abstract screening resulted in a total of 24 articles that were eligible for full-text screening. From the references of these 24 records, a citation search yielded a further 12 articles, of which only one record was eligible for full-text screening. Thus, 25 records were included in the full review, of which 11 records were included in the data extraction (see: Flow Chart, Figure 1). Out of the 11 included studies, 45% (*n* = 5) were from databases, 45% (*n* = 5) from expert recommendations and 10% (*n* = 1) from citation searching. In total, 73% (*n* = 8) of the studies are in German.

### Study characteristics

Three studies are randomized controlled trials (RCT), six studies are of cross-sectional design and two studies are qualitative studies. Among the included studies, five were published via peer-reviewed journals and four are scientific theses, both at bachelor’s and master’s level. The years of publication range from 2016 to 2024. According to our inclusion criteria of a population of at least 50% farmers within green professions, farmers represent the main study population of the included studies. One study included only farmers as their study population (32). Another study investigated trainees of green professions (33). Six studies assessed farmers together with horticulturists (34) or farmers together with horticulturists and foresters, i.e., green professions (35–39). The two qualitative studies interviewed farmers and, additionally, the wife of a farmer (40) as well as consultants being involved in farm successions (41).

The two qualitative studies investigated seven and eight individuals and the broad range of individuals included within the quantitative studies goes from n = 81 to 5,488. Only seven out of the 11 included studies reported the mean age of the study participants, but not all of them also reported the age range or standard deviation. The reported mean values were between 20.13 and 56.98 years. One study gave no indication about the age of study participants (41) and the remaining studies provided some limited details such as only reporting the age range. A similar pattern is presented regarding gender of the included participants. Two studies do not mention the distribution of gender of their study participants (37, 41). One study included only women (42). There are only two studies where less men participated than women (35, 36). Furthermore, in one study, data collection was also done among farmers living in Austria (corresponding to 15% of the study sample) (32).

### Results of studies

Nine out of the eleven included studies report outcomes related to mental health (32, 33, 35–40, 42). Although the remaining studies dealt with the concept of mental health, they did not report any direct outcomes, instead they described stressors. Six of the eleven included studies analyse these stressors in agriculture in connection with mental health (34, 37, 39–42).

Table 1 gives an overview of the key information for the included studies, covering study sample information (especially, if there is a selective study sample) and (if possible) the recruitment strategies with eligibility criteria. The result section only states values, which were reported by the authors of the original studies. If values such as frequencies, mean or standard deviation are not reported within the result section, this is due to a lack of presentation in the included studies. The results of the individual studies are also reported using the terminology used by the authors of the original studies (green professions or farmers). In Table 2, there is a more detailed presentation of assessed measurements. The missing details in Tables 1, 2 also reflect the overall heterogeneity and limited methodological transparency observed across the included studies, which should be considered when interpreting the findings.

**Table 2:** Overview of the measurements related to mental health outcomes in the included studies.

Reference nr.	Author(s)	Publication year	Measurement	Source of measuring instrument	Number of items	Scale description (Information mentioned in the included studies)
(32)	Roth	2021	Hospital Anxiety and Depression Scale (HADS-D)	Herrmann-Lingen, C., Buss, U. and Snaith, R. P. (2011). Hospital Anxiety and Depression Scale – Deutsche Version (HADS-D). Bern: Hans Huber.	14 (7 Anxiety and 7 Depression)	0–21 range; 0–42 total value no symptoms (0–7) mild symptoms (8–10) severe symptoms (11–14) very severe symptoms (15–21) score of >11 (severe & very severe)
(32)	Roth	2021	Copenhagen Burnout Inventory (CBI)	Nübling, M., Stößel, U., Hasselhorn, H. M., Michaelis, M. and Hofmann, F. (2005). Methoden zur Erfassung psychischer Belastungen – Erprobung eines Messinstrumentes (COPSOQ). Bremerhaven: Wirtschaftsverlag NW (Schriftenreihe der Bundesanstalt für Arbeitsschutz und Arbeitsmedizin Fb 1,058). Kristensen, T., Borritz, M., Villadsen, E. and Christensen, K. (2005). The Copenhagen Burnout Inventory: A new tool for the assessment of burnout. Work & Stress 19(3), 192–207. doi:10.1080/02678370500297720	6	Personal Burnout; 0-100 range; burnout cut-off: four of six items must be over 75
(33)	Betz, Engemann and Vu	2022	Pittsburgh Sleep Quality Index (PSQI)	No information provided	No information provided	No information provided
(33)	Betz, Engemann and Vu	2022	Epworth Sleepiness Scale (ESS)	No information provided	No information provided	No information provided
(33)	Betz, Engemann and Vu	2022	WHO-5 Well-Being Index (WHO-5)	No information provided	No information provided	No information provided
(33)	Betz, Engemann and Vu	2022	Azubi-Gesundheitsfragebogen (AGF)	No information provided	No information provided	No information provided
(35)	Braun et al.	2021	Quick Inventory of Depressive Symptomology (QIDS-SR16)	Rush, A. J., Trivedi, M. H., Ibrahim, H. M., Carmody, T. J., Arnow, B., Klein, D. N., Markowitz, J. C., Ninan, P. T., Kornstein, S., Manber, R., Thase, M. E., Kocsis, J. H., Keller, M. B., 2003. The 16-Item Quick Inventory of Depressive Symptomatology (QIDS), Clinician Rating (QIDS-C), and Self-Report (QIDS-SR): a Psychometric Evaluation in Patients with Chronic Major Depression. Biol Psychiatry 54, 573–583. https://doi.org/10.1016/S0006-3223(03)01866-8.	16	0-27 total score range; Each item: 4-point scale, rated from 0 to 3 with a higher score indicating a more severe depressive symptomology Classified symptoms: normal (0–5) mild (6–10) moderate (11–15) severe (16–20) very severe (20–27)
(35)	Braun et al.	2021	The Perceived Stress Scale (PSS)	Cohen, S., Kamarck, T., Mermelstein, R., 1983. A Global Measure of Perceived Stress. J. Health Soc. Behav. 24, 385–396. https://doi.org/10.2307/2136404.	10	0-40 total score range
(35)	Braun et al.	2021	Insomnia Severity Index (ISI)	Bastien, C. H., Vallières, A., Morin, C. M., 2001. Validation of the Insomnia Severity Index as an outcome measure for insomnia research. Sleep Med 2, 297–307. https://doi.org/10.1016/S1389-9457(00)00065-4.	7	0-28 total score range
(35)	Braun et al.	2021	Generalized Anxiety Disorder (GAD-7)	Spitzer, R. L., Kroenke, K., Williams, J. B. W., Löwe, B., 2006. A Brief Measure for Assessing Generalized Anxiety Disorder: the GAD-7. Arch. Intern. Med. 166, 1,092–1,097. https://doi.org/10.1001/archinte.166.10.1092.	7	0-21 total score range
(35)	Braun et al.	2021	Composite International Diagnosis Interview (CIDI)	Auerbach, R. P.; Mortier, P.; Bruffaerts, R.; Alonso, J.; Benjet, C.; Cuijpers, P.; Demyttenaere, K.; Ebert, D. D.; Green, J. G.; Hasking, P.; et al. WHO World Mental Health Surveys International College Student Project: Prevalence and Distribution of Mental Disorders. J. Abnorm. Psychol. 2018, 127, 623–638.	No information provided	Self-report version
(35)	Braun et al.	2021	Panic and Agoraphobia Scale (PAS)	Bandelow, B., 1995. Assessing the efficacy of treatments for panic disorder and agoraphobia. II. The Panic and Agoraphobia Scale. Int. Clin. Psychopharmacol. 10, 73–81 https://doi.org/10.1097/00004850-199506000-00003. Bandelow, B., Hajak, G., Holzrichter, S., Kunert, H. J., Rüther, E., 1995. Assessing the efficacy of treatments for panic disorder and agoraphobia. I. Methodological problems. Int. Clin. Psychopharmacol. 10, 83–93. https://doi.org/10.1097/00004850-199506000-00004.	13	0-52 total score range
(35)	Braun et al.	2021	Maslach-Burnout Inventory General Survey (MBI-GS)	Maslach, C., Jackson, S. E., Leiter, M. P., 1997. Maslach Burnout Inventory. In: Zalaquett, C. P., Wood, R. J. (Eds.), Evaluating Stress: A Book of Resources. The Scarecrow Press, pp. 191–218.	5	Emotional exhaustion - 7-point Likert scale (no range description); mean value and standard deviation (M (SD))
(35)	Braun et al.	2021	Assessment of Quality of Life (AQoL-8D)	Richardson, J., Iezzi, A., Khan, M. A., Maxwell, A., 2014. Validity and Reliability of the Assessment of Quality of Life (AQoL)-8D Multi-Attribute Utility Instrument. Patient 7, 85–96. https://doi.org/10.1007/s40271-013-0036-x.	35	No information provided
(36)	Braun et al.	2022	Quick Inventory of Depressive Symptomology (QIDS-SR16)	Rush, A. J., Trivedi, M. H., Ibrahim, H. M., Carmody, T. J., Arnow, B., Klein, D. N., Markowitz, J. C., Ninan, P. T., Kornstein, S., Manber, R., Thase, M. E., Kocsis, J. H., Keller, M. B., 2003. The 16-Item Quick Inventory of Depressive Symptomatology (QIDS), Clinician Rating (QIDS-C), and Self-Report (QIDS-SR): a Psychometric Evaluation in Patients with Chronic Major Depression. Biol Psychiatry 54, 573–583. https://doi.org/10.1016/S0006-3223(03)01866-8.	16	No information provided
(36)	Braun et al.	2022	Composite International Diagnosis Interview (CIDI)	Auerbach, R. P.; Mortier, P.; Bruffaerts, R.; Alonso, J.; Benjet, C.; Cuijpers, P.; Demyttenaere, K.; Ebert, D. D.; Green, J. G.; Hasking, P.; et al. WHO World Mental Health Surveys International College Student Project: Prevalence and Distribution of Mental Disorders. J. Abnorm. Psychol. 2018, 127, 623–638.	No information provided	Self-report version
(36)	Braun et al.	2022	Generalized Anxiety Disorder (GAD-7)	Spitzer, R. L., Kroenke, K., Williams, J. B. W., Löwe, B., 2006. A Brief Measure for Assessing Generalized Anxiety Disorder: the GAD-7. Arch. Intern. Med. 166, 1,092–1,097. https://doi.org/10.1001/archinte.166.10.1092.	7	No information provided
(36)	Braun et al.	2022	The Perceived Stress Scale (PSS)	Cohen, S., Kamarck, T., Mermelstein, R., 1983. A Global Measure of Perceived Stress. J. Health Soc. Behav. 24, 385–396. https://doi.org/10.2307/2136404.	10	No information provided
(36)	Braun et al.	2022	Insomnia Severity Index (ISI)	Bastien, C. H., Vallières, A., Morin, C. M., 2001. Validation of the Insomnia Severity Index as an outcome measure for insomnia research. Sleep Med 2, 297–307. https://doi.org/10.1016/S1389-9457(00)00065-4.	7	No information provided
(36)	Braun et al.	2022	Assessment of Quality of Life (AQoL-8D)	Richardson, J., Iezzi, A., Khan, M. A., Maxwell, A., 2014. Validity and Reliability of the Assessment of Quality of Life (AQoL)-8D Multi-Attribute Utility Instrument. Patient 7, 85–96. https://doi.org/10.1007/s40271-013-0036-x.	No information provided	No information provided
(37)	Schöllhorn	2020	Perceived stress was assessed with eight items (original scale unknown)	Blankenstein, Gassner, Hilken und Milz (year unknown)	8	1–5 range; higher values indicate higher perceived stress
(37)	Schöllhorn	2020	Standardised questionnaire for stressors (original scale unknown)	Unknown	38	Not provided
(38)	Stier-Jarmer et al.	2020	Perceived Stress Questionnaire (PSQ)	Fliege, H.; Rose, M.; Arck, P.; Levenstein, S.; Klapp, B. F. Validierung des “Perceived Stress Questionnaire“ (PSQ) an einer deutschen Stichprobe. Diagnostica 2001, 47, 142–152. Fliege, H.; Rose, M.; Arck, P.; Walter, O. B.; Kocalevent, R.-D.; Weber, C.; Klapp, B. F. The Perceived Stress Questionnaire (PSQ) Reconsidered: Validation and Reference Values From Di_erent Clinical and Healthy Adult Samples. Psychosom. Med. 2005, 67, 78–88.	No information provided	Subscales M (mean) and standard deviation (SD): Worries, Tension, Joy, Demands; sum score; n (%): Stress level normal (≤45) Stress level increased (46–59) Stress level high (≥60)
(38)	Stier-Jarmer et al.	2020	Burnout symptoms, assessed with Maslach Burnout Inventory General Survey (MBI-GS-D)	Büssing, A.; Glaser, J. Managerial Stress und Burnout, A Collaborative International Study (CISMS), Die Deutsche Untersuchung; Technische Universität, Lehrstuhl für Psychologie: München, Germany, 1998.	No information provided	Emotional Exhaustion: mean value and standard deviation (M (SD)); n (%): No burnout (<3.6 points) Risk of burnout (3.6–5.0 points) Symptoms of burnout (>5.0 points)
(38)	Stier-Jarmer et al.	2020	Sleep disorders, assessed with Insomnia Severity Index (ISI)	Gerber, M.; Lang, C.; Lemola, S.; Colledge, F.; Kalak, N.; Holsboer-Trachsler, E.; Pühse, U.; Brand, S. Validation of the German version of the insomnia severity index in adolescents, young adults and adult workers: Results from three cross-sectional studies. BMC Psychiatry 2016, 16, 1–14.	No information provided	n (%): Absence of insomnia (<8 points) Sub-threshold insomnia (8–14 points) Moderate insomnia (15–21 points) Severe insomnia (>21 points)
(38)	Stier-Jarmer et al.	2020	Depression, assessed with Patient Health Questionnaire (PHQ-2)	Löwe, B.; Kroenke, K.; Grafe, K. Detecting and monitoring depression with a two-item questionnaire (PHQ-2). J. Psychosom. Res. 2005, 58, 163–171.	2	Sum score; 0–6 range, 3 as cut-off
(38)	Stier-Jarmer et al.	2020	Anxiety, assessed with Generalized Anxiety Disorder (GAD-2)	Kroenke, K.; Spitzer, R. L.; Williams, J. B.; Monahan, P. O.; Lowe, B. Anxiety disorders in primary care: Prevalence, impairment, comorbidity, and detection. Ann. Intern. Med. 2007, 146, 317–325.	2	Sum score; 3 as cut-off
(38)	Stier-Jarmer et al.	2020	Well-being, assed with the World Health Organization 5-item Well-Being Index (WHO-5)	Bech, P. Measuring the Dimension of Psychological General Well-Being by the WHO-5. Qual. Life Newsl. 2004, 32, 15–16. Bech, P.; Olsen, L. R.; Kjoller, M.; Rasmussen, N. K. Measuring well-being rather than the absence of distress symptoms: A comparison of the SF-36 Mental Health subscale and the WHO-Five well-being scale. Int. J. Methods Psychiatr. Res. 2003, 12, 85–91.	5	No specific information provided
(38)	Stier-Jarmer et al.	2020	Health status, EuroQol (EQ-5D-5L)	Rabin, R.; de Charro, F. EQ-5D: A measure of health status from the EuroQol Group. Ann. Med. 2001, 33, 337–343. Herdman, M.; Gudex, C.; Lloyd, A.; Janssen, M.; Kind, P.; E Parkin, D.; Bonsel, G. J.; Badia, X. Development and preliminary testing of the new five-level version of EQ-5D (EQ-5D-5L). Qual. Life Res. 2011, 20, 1727–1736.	5	Health Status M (SD); no specific information provided Reported - n (%): Mobility: no problems Self-care: no problems Usual activities: no problems Pain/discomfort: no problems Anxiety/depression: no problems
(39)	Hetzel et al.	2016	Life satisfaction (LEZU)	Diener E, Emmons RA, Larsen RJ et al. The Satisfaction with Life Scale. J Person Assess 1985; 49: 71–75 Glaesmer H, Grande G, Braehler E et al. The German version of the Satisfaction with Life Scale (SWLS): psychometric properties, validity, and population-based norms. European J Psych Assess 2011; 27: 127–132	5	7 response levels (no range description); High scores correspond to high values
(39)	Hetzel et al.	2016	Mental health impairments (PSYC)	Bakker AB, Demerouti E. The job demands-resources model: state of the art. J Manag Psychol 2007; 22: 309–328	5	5 response levels (no range description); High scores correspond to high values
(40)	Buhne	2019	Qualitative study; burnout and symtpoms related to burnout	No information provided	No information provided	No information provided
(42)	von Davier	2024	Copenhagen Burnout Inventory (CBI)	Kristensen, T., Borritz, M., Villadsen, E. and Christensen, K. (2005). The Copenhagen Burnout Inventory: A new tool for the assessment of burnout. Work and Stress 19(3), 192–207. doi:10.1080/02678370500297720 Stöbel-Richter Y, Daig I, Brähler E, Zenger M (2013) Prävalenz von psychischer und physischer Erschöpfung in der deutschen Bevölkerung und deren Zusammenhang mit weiteren psychischen und somatischen Beschwerden. PPmP 63(03/04): 109–114, zu finden in <https://www.thieme-connect.com/products/ejournals/abstract/10.1055/s-0032-1331704 > [zitiert am 02.4.2024]	6	Personal burnout; 0–100 range; 62.5 as cut-off for burnout risk

### Mental health outcomes

The nine studies reporting mental health outcomes have used different instruments, making comparisons possible only to a limited extent. To give an overview on the instruments used, we provide a detailed description in Table 2 where we report the used questionnaires and, if specified, their scale calculations and description. For the following overview of results, we have categorized the observed findings into subheadings and show similar concepts together. We report findings transparently, but need to mention that almost all of the included studies lack relevant information for evaluating the results.

### Depressive symptoms

Six studies showed results for depressive symptoms and/or symptom severity (32, 35, 36, 38), well-being (33, 38) or psychological complaints (39). Two studies further assessed mental health care service use and onset or remission of major depressive disorder (35, 36).

One randomized controlled trial RCT investigated telephone coaching sessions in employees of green professions (*n* = 108; e.g., 65.7% had animal husbandry), who show elevated stress levels and are at risk of developing (mental) health impairments (38). According to the authors, 24.1% of participants reported symptoms of depression, which would require further professional investigation (38). Another study among farmers in Germany and Austria (15% of the study population are farmers in Austria) assessed depressive symptoms among 2,788 participants with the German version of the Hospital Anxiety and Depression Scale (HADS-D) (32). For depressive symptoms, 23.6% of participants showed values indicating a clinically relevant depression (32).

One pragmatic RCT investigated effectiveness of internet-based interventions to prevent depression in green professions (35). Several stress-related outcomes were assessed at baseline (and a certain level of symptoms were assessed as inclusion criteria, see Table 1). For example, 42.9% of the participants at baseline reported a moderate to very severe level of depressive symptom severity, assessed with the Quick Inventory of Depressive Symptomology (QIDS-SR16). On average, participants reported a mean value of 10.01 (SD = 4.42) indicating a mild level of depressive symptom severity (35). The authors further reported mental health care service use and showed that 5% of the participants were searching for a psychotherapist or were on a waitlist for psychotherapy, at baseline (35).

Another pragmatic RCT from the same group of authors investigated effectiveness of online-based acceptance and commitment therapy to improve chronic pain-associated disability in green professions (36). Baseline data are only shown separately for intervention and control group. The mean depressive symptom severity, also assessed with QIDS-SR16, was reported as 7.63 (SD = 3.80) and 7.89 (SD = 4.43) for intervention and control groups respectively, indicating mild symptoms (36). Regarding mental health care service use, 2.3% in the intervention group, and 2.6% in the control group, are treated by a psychotherapist and 4.7 and 2.6%, respectively, see a psychiatrist, neurologist, or psychosomatic medicine specialist (36). No information was provided on the comparison of the mental health care service use in the general population, so it is not possible to classify the results.

Two studies show results for (mental) well-being using the World Health Organization well-being index (WHO-5) (33, 38). Among green professions who reported increased stress levels as an inclusion criterion for study participation, the reported average value for well-being at baseline was 45.0 (SD = 19.1) (38). A cut-off of 50 is usually used when the WHO-5 is applied as a screening tool for depression, and values less than 50 indicate the possible presence of depression (43). Betz et al. (33) reported that among trainees in green professions, 23% of participants reported to have impaired well-being, which would be a better finding compared to a similar aged population.

Another study investigated the health status of older individuals working in green professions (39). Psychological complaints were assessed with five items ranging from one to five (higher numbers indicating more complaints) and a mean value of 2.43 (SD = 1.01.) was shown (39). This value also proved to be low compared to other occupational sectors such as commercial and technical fields (39).

### Anxiety symptoms

Four studies assessed generalized anxiety symptoms (32, 35, 36, 38). Three of these have used the generalized anxiety disorder questionnaire in different length-version (GAD-7 and GAD-2), while Roth (32) assessed anxiety with the HADS-D.

A mean value of 2.5 (SD = 1.5) was reported for anxiety in the study by Stier-Jarmer et al. (38). According to the authors, 39.2% had symptoms of anxiety, which would require further professional examination. Similarly, Roth reported that 32.5% of participants showed values indicating a clinically relevant anxiety disorder (32). Braun et al. (35) showed average levels at baseline only separately for intervention and control group. Mean values of 7.84 (SD = 4.39) and 8.09 (SD = 3.47) were reported for intervention and control group. The same group of authors also reported only separate baseline mean values for the study participants in another RCT, showing anxiety mean values of 7.16 (SD = 4.08) and 5.87 (SD = 4.08) for intervention and control group (36). A cut-off of ten is usually applied to identify clinically relevant generalized anxiety disorders, and values greater than 5 can indicate mild levels of anxiety symptoms (44). These results indicate that both the intervention and the control group show symptoms of an anxiety disorder.

### Burnout symptoms

Burnout symptoms were investigated in one qualitative study (40). Personal burnout, i.e., not addressing the work context, was assessed by two studies (32, 42). Further two studies assessed burnout via emotional exhaustion (35, 38).

The qualitative study aimed to investigate how farmers perceive the development, progression and treatment of burnout. This study assessed several symptoms regarding burnout among six farmers and the wife of a farmer who had committed suicide, who was not further differentiated in the study from other farmers (40). After receiving treatment for burnout, the return-to-work process was described as a great challenge for farmers. Compared to other occupational sectors, farmers report that they cannot go back to work with reduced working hours, but have to start fulltime again in order to prevent further economic difficulties (40). They have to learn other strategies, such as delegating their work tasks and taking time for breaks.

Two studies used the scale personal burnout out of the Copenhagen Burnout Inventory (CBI) (32, 42). It was reported that 27% of farmers in Germany and Austria are affected by burnout (32). Differentiated into women and men, it was shown that 23.2% of men and 37.2% of women are affected by burnout (32). Significant differences were found for burnout or other mental health outcomes in relation to gender or age. Women, for example, reported to have more often clinically relevant anxiety symptoms and burnout than expected (32). Further, Roth (32) investigated possible differences for different forms of farming and reported that dairy farmers were more often affected by depression, anxiety or burnout. One study investigating only women who live or work in farms also assessed burnout with the CBI (42). Out of the participants, 21.4% were at risk of having burnout.

Two studies assessed emotional exhaustion with different versions of the Maslach Burnout Inventory General Survey (MBI-GS-D) (35, 38). Of the individuals in green professions with increased stress levels at baseline of an RCT, 72.2% had either risk of burnout (57.4%, indicating values from 3.6 to 5) or symptoms of burnout (14.8%, indicating values >5) (38). Mean values for emotional exhaustion of both studies differed and due to lack of reported data on number of items and scale range, a comparison or classification of these values is not possible (35, 38).

### Sleep disorders

Five studies investigated sleep disorders (33, 35, 36, 38, 40). Three of them assessed insomnia (35, 36, 38), while one qualitative study reported sleep disorders or fatigue (40) and another study reported sleep problems and daytime sleepiness (33).

The qualitative study of Buhne (40) reported that having sleep disorders resulted in being tired at work. Betz et al. (33) questioned trainees of green professions and reported that 28% of participants have a poor sleep quality. This was assessed with the Pittsburgh Sleep Quality Index (PSQI) and Epworth Sleepiness Scale (ESS). One third of the participants reported to have experienced micro sleep while driving, resulting in an accident for 6% of these participants (33).

Three studies assessed insomnia with the Insomnia Severity Index (ISI) (35, 36, 38). Stier-Jarmer et al. (38) reported that 35% of the participants had moderate or severe insomnia. Braun et al. (35) reported separate mean values of 9.96 (SD = 5.19) and 11.22 (SD = 5.21) for intervention and control group at baseline. Braun et al. (36) reported mean values at baseline of 8.63 (SD = 4.91) and 9.08 (SD = 6.16) for intervention and control group for insomnia. Values 8–14 for example indicate subthreshold insomnia and values greater than 14 indicate clinically relevant insomnia (45).

### General health status

Two studies have assessed the general health status (33, 38). For example, in a study among trainees of green professions, 78% of the participants reported to have a good or very good general health status (33). The most common health complaints were back pain and headaches (33).

### Perceived stress

Perceived stress was assessed by four studies (35–38).

In one study with 634 participants belonging to green professions, perceived stress was assessed with eight items of a questionnaire not identified (37). It was reported that participants aged between 50 and 65 reported the highest stress levels. Women reported higher stress levels than men and the lowest stress levels were observed for farmers without a formal agricultural qualification (37).

In the RCT among stressed individuals in green professions, participants reported to have an average value of 55.3 (SD = 14.6) for the Perceived Stress Questionnaire (PSQ) at baseline, showing an increased level of stress (38). The authors also stated that nearly 74% of the participants had either increased (values from 46 to 59) or high (values ≥60) stress levels, according to the PSQ (38).

In the two pragmatic RCTs, perceived stress was assessed with the perceived stress scale (35, 36). An average value of 20.61 (SD = 6.80) was shown in the intervention group and a mean value of 22.21 (SD = 5.93) was shown in the control group at baseline in the RCT about effectiveness of an internet-based intervention to prevent depression, which corresponds to an intermediate perception of stress (35). In the other pragmatic RCT about effectiveness of an online-based therapy to improve chronic pain-associated disability, the participants also started at baseline with mean values of 18.26 (SD = 6.60) and 17.76 (SD = 6.94), corresponding to an intermediate perceived stress level (36).

### Stressors mentioned alongside mental health

Based on the six studies which dealt with stressors related to mental health, seven overarching categories of psychological stressors were identified: (1) environmental and climate-related stressors, (2) economic stressors, (3) bureaucratic and regulatory stress factors, (4) societal challenges, (5) social and family challenges, (6) personal stressors and (7) working conditions (34, 37, 39–42). In addition to these identified categories, Schöllhorn (37) also points out that the perception and rating of the various stress factors differs between women and men in agriculture. Women tend to feel more stressed by conflicts within the family and financial problems of the farm, whereas men are mainly overwhelmed by bureaucracy and high requirements and restrictions (37).

#### Environmental and climate-related stressors

Among the six studies that analysed stressors in agriculture, one cross-sectional study surveyed farmers on climatic stressors (37). It found that farmers over the age of 65 in particular but also part-time farmers and farmers who practise organic farming, perceive fears of storms as stressful. Fears of animal diseases tend to play a minor role and working with animals in general does not appear to be a stressor for farmers.

#### Economic stressors

Economic pressure, liquidity problems and the associated fears for the future are investigated by four studies as economic stressors (37, 40–42). Difficulties to calculate investment costs, high investment costs as well as the often generally high economic value of farms put farmers under pressure (37, 40, 41) and lead to fears for the future (37). The qualitative results of Greiner (41) emphasize these aspects as a burden for young farmers in particular. Buhne (40) states that business farming is leading to increased competition among farmers, with a loss of collegiality (40). The study by von Davier (42) on mental health of women in agriculture showed that women who see the existence of the farm threatened are significantly more at risk of burnout than women in agriculture who assume a better future for the farm. Uncertainty regarding organization of large financial investments and a poor personal income situation are also described by the women as stressful (42). Schöllhorn (37) also points out that farmers only feel stressed by low profitability and high interest rates (borrowing costs) when liquidity problems arise.

#### Bureaucratic and regulatory stress factors

Four studies describe the burden of bureaucracy as an important stress factor in agriculture (34, 37, 40, 41). The study by Schöllhorn (37) identifies bureaucracy as the greatest mental stress factor in agriculture. The legal frameworks to which agriculture is subjected to makes farmers feel externally determined and dependent (34, 40). This is because the sensibility and practicability of policies is not questioned by the political side (according to the farmers’ perception) and makes profitable farm management almost impossible (40). Further, Knoop and Theuvsen (34) emphasizes that compliance is monitored, which also places a mental burden on farmers. Schöllhorn (37) found that the mental burden of bureaucracy is a key cause of sleep deprivation, especially on livestock farms. In addition, attempts to keep the farm profitable, such as direct marketing, are in turn associated with a high mental burden for farmers (37). Schöllhorn (37) also finds that individuals aged from 35 to 65 in particular, for whom farming is their main occupation, feel burdened by bureaucracy. Women, on the other hand, hardly reported any burden from bureaucracy (42).

#### Societal challenges

Two of the six studies that report results on psychological stressors in agriculture (40, 42) show that farmers feel stressed by a perceived negative social image of agriculture. According to von Davier (42), women who are burdened by the social image of agriculture are more at risk of burnout than other women in the agricultural sector. Women from organic farms are less stressed by the social image of agriculture than women from conventional farms. Women from conventional farms are most affected by the poor social image of agriculture (42). Farmers in the qualitative study by Buhne (40) name society’s unobjective and emotionally driven debate about agriculture as well as society’s desire for cheap food without realising the production effort behind it as reasons for this poor image.

#### Social and family challenges

All six studies describe stressors regarding the social or family environment in agriculture (34, 37, 39–42). Hetzel (39) and Greiner (41) describe the generational change on farms as a decisive challenge and emotional burden, which always leads to conflicts and considerable disputes, for example when the older generation is unable to let go, is not in favour of modernisation driven by the younger generation or holds different values. Greiner (41) found that formal aspects, such as contracts or arrangements for caring for the older generation, also play an important role in the increased perception of stress when handing over a farm. Ultimately, this can also lead to emotionally stressful conflicts between couples and personal roles (41). Therefore, it is not surprising that most of the stress in the age group of 20 to 34 years is felt through conflicts within the family (34, 37). However, according to Hetzel (39), Schöllhorn (37) and von Davier (42), generational conflicts are less stressful for men than for women. One possible reason described by Buhne (40) is the unpaid work on the farm by family members, which exacerbates family conflicts.

#### Personal stressors

Various personal factors appear to increase the perception of stress in agriculture. The number of children seems to have an impact on the stress perception of women in agriculture, as women without children or with one child have a lower risk of burnout than women with more than one child under the age of six (42). Another stressor mentioned by women is the remaining high workload around childbirth, resulting in perception of tension between work and childcare as a consequence of this double burden (42). According to Buhne (40), farmers do not prefer to work with employees not being family members because it is emotionally difficult to engage with them. Next to the (poor) rating of their own health, the own role on the farm, e.g., being the wife of the farm owner, also leads to a higher risk of burnout among women in agriculture (42). This detailed information in connection with the life situation and overall health status is not described in the included studies for men or rather in connection with economic and bureaucratic stressors.

#### Working conditions

Four of the six studies that analyse stressors in agriculture describe stressors that result from the specific working conditions in the sector (34, 37, 40, 42). Two studies describe the dissolution of boundaries between private and work life as stressful due to the fact that both aspects are happening in the same place (34, 40). Personal needs, hobbies, time with the family and holidays are less prioritized than the farm (34, 40) and are a particular burden for farmers with an average age of 40 (34). Other stressful working conditions identified by Knoop and Theuvsen (34), Schöllhorn (37) and Buhne (40) are the long working days, the time pressure and the intensity of work and variety of tasks (37).

## Discussion

This scoping review aimed to explore which mental-health outcomes are reported among farmers in Germany and which potential implications are resulting from the findings. Our knowledge synthesis of the literature revealed that only few studies assessed mental health of farmers in Germany with, in some cases, poor descriptions, e.g., of the study population or the measuring instruments used, resulting in a total of only eleven included studies, out of which five were found through additional expert sources. Outcomes examined in the studies were depressive symptoms, anxiety symptoms, burnout symptoms, sleep disorders, general health status or perceived stress. About half of the included studies reported stressors alongside mental health outcomes, such as social and family challenges (i.e., generational conflicts) or difficult working conditions (e.g., time pressure, long working hours, dissolution).

Internationally, several studies show high rates of mental health problems in the agricultural sector (3). Many of these studies focus on depression, stress or suicide, while anxiety or burnout are less frequently investigated, worldwide (3). The studies examined in the present review have also considered anxiety (four studies) and burnout symptoms (five studies) in the German context. Compared to the 12-month prevalence of anxiety disorders (15.3%) in an average German population (46), percentages of anxiety symptoms indicating clinically relevant anxiety disorders were considerably higher among individuals working in agriculture in two of the reviewed studies (32, 38). However, as one of the two studies evaluated a telephone coaching to reduce perceived stress, there is likely a selection bias present, as a higher amount of farmers perceiving stress were included (38). This is likely to be also true for two other randomized controlled trials examining interventions for reduction of depression and chronic pain, which indicated higher symptoms of anxiety in their study populations (35, 36). Although the data is not sufficient to estimate the prevalence for anxiety disorders among German farmers, the high proportion of farmers with anxiety disorders warrant to further study this mental health problem regardless of whether it is more prevalent than in the general population.

Among the studies investigating burnout, one qualitative study reported that farmers who had received treatment for burnout described experiencing great difficulties when returning to work, as a stepwise return-to-work procedure was not feasible and stressors such as financial pressure were perceived as particularly high (40). Another quantitative study showed that 27% of the farmers (living in Germany or Austria) are affected by burnout (32). For example, a 12-month prevalence of a diagnosed burnout-syndrome for a general German population was reported to be 1.5% (47). However, there is need for more studies using similar measurements to be able to compare these prevalences. A similarly high estimation of 21.4% of women being affected by burnout was found for women in the agricultural sector (42). One study among women in agriculture in Switzerland reported a slightly higher prevalence of 15% (48). Therefore, these values should raise concern and underscore the need for more sound and precise investigation of burnout prevalence for this particular occupational sector, especially, as the international evidence is also scarce: A scoping review on the mental health of farmers worldwide identified only two studies (from Canada and Finland each) investigating burnout (3, 49, 50). One study among Irish farmers reported a prevalence rate of 23.6% of burnout (51), pointing in the same direction as the findings for German farmers (32, 42).

For Germany, we found no study exploring outcomes related to suicide among farmers. As several European studies mention increased risks for suicide in the agricultural sector compared to other occupational sectors or general population samples, further research is warranted to generate evidence for Germany (10, 11). For example, in their trend analysis of suicides in France, Bossard et al. (10) describe that the risks of dying from suicides were 28% higher among male farmers, than in the general population in 2008 and in 2009 (22% higher). Factors such as long working hours, high physical demands or high stress due to unforeseen natural events may especially contribute to increased risks for suicides among farmers (4). From the included studies, financial burdens (high investment costs), high bureaucratic requirements, and a lack of work-life balance in particular are identified as burdens for farmers in Germany (34, 37). These are some of the risk factors for suicide that have been already identified in the international literature (4) and indicate a potential risk for German farmers. The particular conditions for farmers need to be considered in future research to detect potential farmers at risk for suicidal ideations.

The most assessed mental health outcome in the included studies was depressive symptoms. Six studies reported findings on depressive symptoms using various measurements. However, many of these studies did not report prevalence rates of symptoms, but were mostly randomized controlled trials. The only study investigating prevalence of depression indicated that 23.6% of participants (farmers either living in Germany or Austria) reported clinically relevant depression symptoms (32). However, the results have to be interpreted carefully, as eligibility criteria were not reported. These numbers would need to be confirmed by studies using representative samples. Internationally, studies reveal a similar pattern of high rates for depression among farmers: One systematic review on mental health among farmers worldwide found 111 studies examining depression (3). Half of these studies were conducted in the US, while Australia, UK and Norway were other countries that investigated mental health among farmers more frequently (3). For example, a study from England and Wales reported that 31% of the surveyed farmers have problems with anxiety/depression (1). Another systematic review and meta-analysis of the same author group included 45 studies on the prevalence of depression among farmers, of which 42 were conducted in the US (26). The meta-analysis was conducted on the centre for epidemiologic studies – depression scale (CES-D), and revealed an overall prevalence of depression of 20% with high heterogeneity, when considering depression as a binary outcome (26). Other reviews also mention that further research is needed due to limited evidence and that more sound science is required with a focus on assessment of depression or other mental health outcomes (52). Due to the reported large heterogeneity, there seem to be relevant methodological or contextual factors contributing to the between-study variance (26). These factors may for example be geographic locations, the type of farming, gender, political changes or factors such as willingness to talk about mental health (26). Half of the included studies within the meta-analysis used convenience sampling, contributing to a risk of selection bias, for example (26). Also, as response rates were often not reported, this may lead to the underestimation of reported prevalence rates as those with depression may be less likely to respond to surveys (26). One study compared farmers with non-farmers or their siblings and showed that farmers had the highest odds of having high depression symptoms (53). For example, farmers had the highest odds of reporting depressive symptoms (Odds Ratio = 1.99, confidence interval 1.55–2.55) compared to higher grade professionals (53). A longitudinal study in Australia among farmers and non-farming workers showed that farmers in rural areas report poorer mental health compared to non-farming workers in rural areas (54). In general, farmers were also less likely to seek health services, such as a general practitioner or a mental health professional than non-farm workers (54). For example, one study among green professions in Germany included in our review reported that only 5% of participants of an internet-based intervention to prevent depression were searching for a psychotherapist or were on a waitlist for psychotherapy at baseline, although almost 43% of participants reported moderate to severe levels of depression symptom severity (35). This may also indicate a mismatch between seeking access and the need for treatment among the group of working farmers. Further, it is possible that existing services are not known among farmers or that they are not accessible or even lacking in rural areas (55).

One of our included studies reported the mean value of the WHO-5 questionnaire, assessing mental depressive symptoms (38). Values below 50 can indicate a depression. For a representative sample of the general population in Germany, there is normative mean data on the WHO-5 questionnaire available for different age groups ranging from 66.80 (SD = 20.52) for individuals being 61 or older to 73.44 (SD = 19.19) for individuals being 40 or younger (56). Similar values are available for the Danish general population, where a population mean score is 70 (43). In the included study among green professions with increased stress levels at baseline, the reported mean value was 45.0 (SD= 19.1) (38) (38). However, that study aimed at reaching farmers who perceive increased stress levels and is thus not reflecting the general farmer population in Germany (38), which is why the results should be interpreted carefully. Another study on Norwegian farmers showed that almost 35% of participants would need further screening for major depression, also assessed with WHO-5 (57), highlighting the need for increased efforts to improve the use of mental health systems and identification of farmers at risks. Almost all of the mentioned studies find notable gender differences, where females show poorer mental health than males, indicating the need for considering gender as relevant determinants in research. This is also shown in the description of the perceived work-related stressors by von Davier (42).

Next, to the depressive symptoms, sleep disorders were another common outcome examined in the studies included in this review (*n* = 5). Sleeping disorders or poor sleep quality may be especially concerning among farmers, as farm work often entails operating heavy machinery or dealing with livestock or pesticides (51). Experiencing sleep problems is associated with poorer mental health (58) and may increase the risk for work stress (51). One study among farmers reported that half of the participants reported poor sleep (51). Among trainees of green professions, a study included in this review, reported that almost one third had poor sleep quality (33). Among adult farmers, insomnia was reported among 35% of participants in one included study, for example (38). Thus, sleeping problems seem to occur frequently among farmers, and should receive more emphasis in national and international research, as their consequences can be harmful for farmer’s health and mental health. One recently published study reported differences for the cold and warm season among farmers and their partners in Switzerland and showed that sleep quality was perceived as better during the cold season (59). This may be related to the fact that harvest time, and thus the peak working period for farmers, takes place in the summer. However, in contrast to the above-mentioned studies, findings of that new cohort study generally reported good sleep quality in both seasons (59). So, there is still a need for research about the sleep quality of farmers, including in relation to their seasonal workload.

One study, included in this review, reported that among trainees of green professions, the general health status was perceived as good or very good for 78% of the participants (33). Something similar was observed among farmers and their partners in Switzerland, where younger farmers report overall better physical health than older farmers (59). Not only for farmers, but also in general, there is a trend observable that physical health is decreasing with age, while mental health is more concerning at lower ages (59–61). In light of our results, this highlights the need for further research to investigate the sources for potentially increased mental health problems among younger farmers. There may be increased economic or digital stressors, for example. However, older farmers may have different views or values on mental health and their subjective well-being compared to younger farmers, indicating potential discrepancies between the generations.

In our review, four studies reported results on perceived stress. Similar to mental health research (see above), females did more often report higher stress levels than males. Mean levels of the perceived stress scale in two studies of this review (35, 36) were reported as intermediate and similar to perceived stress levels, reported in a study among Canadian farmers (62). These findings may show similarity of perceived stress levels among farmers in different countries; however, more research would be needed to confirm this suggestion.

Next to the mental health outcomes that were assessed in this review, many of the included studies also assessed stressors in relation to mental health. In our review, these were mainly climate-related stressors, bureaucratic or regulatory stressors, economic stressors, social or family challenges, personal stressors or working conditions. Some of these are frequently examined in the international literature. For example, financial difficulties, government policies or regulations, climate variabilities, role conflicts within the family or time pressure were named in a systematic review on key risk factors for affecting mental health of farmers, globally (2). Further, the farmers mentioned poor physical health as important factors as well as pesticide exposure (2). Other stressors frequently mentioned among farmers in Eastern North Carolina were weather, taxes, being concerned about the future of the farm or problems with machinery (63).

We found that a wide range of topics covered in the international literature were also addressed or touched upon in the studies included in this scoping review. When comparing the findings on the mental health of German farmers with the international literature, cultural and political differences between countries should be taken into account when interpreting the results. This also includes differences in the structure of agricultural businesses, gender roles and social security or insurance systems, which can influence the perception of stress and access to psychological services (7, 26, 42).

### Limitations

This study has some limitations that we would like to address. Due to the nature of this scoping review, we did not conduct a quality assessment for the included studies. However, we believe it is important to mention that many of the studies had methodological shortcomings that came to our attention while screening the articles and we tried to transparently show some of these in our detailed Table 2 of assessed scales for mental health. Due to the inclusion of RCTs or other interventional studies a selection bias is likely present where participants with poorer mental health are included than in a general farming population. Further, there were two studies where it was not clearly reported if the underlying study samples were different from each other (35, 36). Our findings can only give a first overview on mental health among farmers in Germany and cannot be generalized onto other countries or population groups. Nevertheless, compared to other countries worldwide, there are initial indications that mental health problems among German farmers appear to be similarly important. We have only included original studies that were published between 2014 and 2024 (based on the realisation that around 2014 publications have increased (2, 3, 26)), indicating that we may have missed findings that were published before 2014. However, these may be less representative for the current mental health status of German farmers. When conducting this scoping review, we used two large and established databases where we assumed to find the most relevant hits for the topic at hand, and we asked experts in the field for further relevant literature (21, 22). However, with this approach, there is a risk of missing relevant studies not identified by the selected databases and the languages used. Further, in Germany farmers are often considered together with other occupations in the green professions. Some of the studies did not provide sufficient information on participant data, which makes it difficult to interpret the results. Therefore, future studies should try to be very specific in their definition of study samples among farmers.

### Implications

There is still a wide research gap when it comes to mental health among farmers. Although many studies nowadays try to develop interventions targeting mental health of farmers, many of these lack an adequate methodological approach. In some of the included studies, the study population, study design, measurement instruments used and analysis methods are not described with the necessary methodological rigour, which is why studies that describe their methodological approach transparently are particularly necessary for a better understanding of farmers mental health. One systematic review on farmer mental health interventions assessed 92 studies with different study designs of which only one considered a control condition during their investigation (64). The results of this scoping review also show that there is currently a lack of concrete epidemiological data on the prevalence of mental illness in Germany, which would be essential for the targeted development and implementation of preventive measures to promote the mental health of farmers. The investigation of work-related stressors in Germany appears to be still in its early stages. However, knowledge in this area is essential for improving working conditions, also in view of the upcoming changes due to climate change, which will transform work in agriculture in particular (20). Thus, more sound evidence is needed when it comes to farmers and their mental health. As international research suggests, farmers are one of the occupational groups with the highest risk for poor mental health (2, 6, 26). Farmers represent a special working population with unique working conditions and contextual factors, which are often not comparable to other working populations (55, 65). In addition, there are cultural and political differences for farmers worldwide in terms of insurance systems, farm sizes and structures, and gender roles (7, 26, 42). Therefore, it is of utmost importance to increase fundamental research as well as applied research on the mental health among farmers in Germany to enable the development of tailored prevention programs and the implementation of policy measures to combat structural stress factors such as economic pressure and administrative burdens.

## Conclusion

Our review has demonstrated that research on mental health of farmers in Germany is scarce. We could not identify studies that assess suicide risks and only a few reported findings on burnout. There is an indication that poor mental health outcomes among farmers in Germany may occur at a similar rate to that found in international studies, but more frequently than in the general population. However, there is a need for epidemiological studies to gain reliable evidence on the prevalence. The stressors identified can provide a basis for targeted preventive interventions.
