# Supplementary material for: Mental health among farmers in Germany: a scoping review
Source: Front Public Health. 2025 Dec 17;13:1701468. doi: 10.3389/fpubh.2025.1701468 (PMC12754006; doi:10.3389/fpubh.2025.1701468)
Supplement: Supplementary file 2 [file Data_Sheet_2.PDF]

Mental health among farmers in Germany: A rapid review

Meike Heming[1], Louisa Scheepers[1], Kira Schmidt-Stiedenroth[1], Peter Angerer[1]

[1]Institute of Occupational, Social and Environmental Medicine, Centre for Health and Society, Faculty of Medicine, Heinrich-Heine University Duesseldorf, Germany

Review information

**Review Title**	Mental Health among Farmers in Germany: A rapid review
**Review Type**	Rapid review
**Review Language**	English
**Reference to any published review protocol**	Not applicable
**Research Questions**	1. What is known about mental health-related outcomes among farmers in Germany?<br>2. Which potential research implications result from the findings?
**Starting Date**	09/2024
**Completion Date (anticipated)**	03/2025
**Data sources**	Web of Science (Core Collection)<br>Google Scholar<br><br>Additional sources (see below)
**Study types to be included**	Original quantitative, qualitative and mixed-methods studies
**Language restrictions**	English<br>German
**Geographical restrictions**	Germany
**Timely restrictions**	2014-2024
**Study population**	Farmers
**Concept**	Mental health
**Context**	Germany
**Stage of review at the time of this submission**	<u>Completed:</u><br>Selection of databases and additional sources<br>Development of the search strategy<br>Preliminary search<br>Piloting of the study selection process<br>Systematic searching of databases<br><u>Not yet completed:</u><br>Identification of further literature using additional sources<br>Export of results into a citation management software<br>Removal of duplicates<br>Title and abstract screening in light of exclusion criteria<br>Full-text screening in light of exclusion criteria<br>Charting the results<br>Data synthesis and summary

Research Team

**Team Leader**	Peter Angerer (PA)<br>peter.angerer@uni-duesseldorf.de
**Team Members**	Meike Heming (MH)<br>meike.heming@uni-duesseldorf.de<br><br>Louisa Scheepers (LS)<br>louisascheepers@uni-duesseldorf.de<br><br>Kira Schmidt-Stiedenroth (KSS)<br>kira.schmidt.stiedenroth@uni-duesseldorf.de
**Institution**	Institute of Occupational, Social and Environmental Medicine<br>Faculty of Medicine<br>Centre for Health and Society<br>Heinrich Heine University Düsseldorf, Germany
**Funding**	No funding was received for this rapid review
**Conflicts of interest**	The authors declare no potential conflicts of interest

Search Strategies

**Web of Science**	((TS=(farm*)) OR TS=(agriculture))<br><br>AND<br><br>(((((TS=(mental health)) OR TS=(depress*)) OR TS=(suicid*)) OR TS=(burnout)) OR TS=(stress)) OR TS=(anxiety)<br><br>AND<br><br>TS=(german*)<br><br>Filters:<br>- Publication date 2014-09-20 – 2024-09-20<br>- English OR German
**Google Scholar**	Landwirt<br><br>+<br><br>„psychische Gesundheit“<br><br>+<br><br>Deutschland<br><br>Filters:<br>- Publication Year: 2014-2024<br>- Sources in German language

**Additional Sources**	• The SVLFG (The German Social Insurance for Agriculture, Forestry and Horticulture) will be consulted to identify further (and in particularly grey) literature<br>• Reference checking of included full-texts

Review Methods

**Review Procedure**	1. Identification of relevant data bases<br>2. Development of the search strategies and search strings<br>3. Systematic searching of selected databases<br>4. Identification and inclusion of further literature using additional sources<br>5. Export of results into citation management software (e.g. Citavi)<br>6. Removal of duplicates<br>7. Export of results into a screening/review software (e.g. Rayyan) and double check for duplicates<br>8. Title and abstract screening for eligibility<br>9. Full text screening for eligibility<br>10. Charting the results<br>11. Data synthesis and summary
**Exclusion criteria**	1. Language of the publication other than English or German<br>2. Publication outside of the specified time period<br>3. No original study<br>4. Population farmers not included in the study<br>5. Concept mental health not included in the study<br>6. Data not collected in Germany
**Inclusion criteria**	1. Language English or German<br>2. Publication within specified time period<br>3. Original study<br>4. Population farmers included in the study<br>5. Concept mental health not included in the study<br>6. Data collected in Germany
**Development of Search Strings**	All search strings were developed according to the PCC framework [1]:<br>Population: farmers – Concept: mental health – Context: Germany
**Title and Abstract Screening**	Title and abstract screening will piloted: 50 abstracts and 5 full-texts will be screened in parallel by two of the three team members [2]. Disagreements will be solved through discussion. The remaining titles and abstracts will be screened by MH, LS and KSS, but not in parallel.
**Full Text Screening**	Full-text screening will be done by MH, LS and KSS. 20% of the full-texts will be additionally screened by different team member [2]. Disagreements will be solved through team discussion until consensus is reached.
**Data extraction**	Data extraction will be done by KSS, who has previous experience in conducting a review [2]. The resulting extracting table will be double-checked by the other team members using a sample.

	Based on the structures suggested in the guidelines for conducting scoping reviews [1] as well as in the JBI Manual for Evidence (chapter for scoping reviews)[3], we will extract:<br>- Date<br>- Author(s) of publication<br>- Year of publication<br>- Source origin/country origin (if other than Germany)<br>- Aims/purpose of the study<br>- Defining characteristics of study participants and sample size (if applicable)<br>- Type of study (e.g. qualitative, quantitative, mixed-methods)<br>- Specific methodology<br>- Setting and context-related information<br>- Concepts used to capture mental health (if applicable)<br>- How outcomes were measured (if applicable)<br>- Key findings<br>- Authors' conclusion<br>- Reviewer's comments<br><br>The comprehensiveness of the data extraction table will be piloted by two reviewers extracting data from 5 papers. Further extraction columns will be added if needed. Disagreements will be solved through discussion within the team until reaching consensus.
**Meta-Analysis**	Not applicable.
**Critical Appraisal**	Not applicable.
**Data synthesis**	Following guidance for conducting and reporting reviews, [1, 4], we will synthesize data as a graphical representation (table, chart or diagram) and/or provide a descriptive summary.
**Publication**	The results of this review are to be published in a peer-reviewed journal relevant to the review's topic.

REFERENCES

1 Peters MDJ, Godfrey CM, Khalil H, et al. Guidance for conducting systematic scoping reviews. *Int J Evid Based Healthc* 2015;13(3):141–46.

2 Heise TL, Seidler A, Girbig M, Freiberg A, Alayli A, Fischer M, Haß W, Zeeb H. Developing the CAT HPPR
- a critical appraisal tool to assess the quality of systematic-, rapid-, and scoping-reviews investigating
interventions in health promotion and prevention [Abstract] (2020). Online available: https://abstracts.cochrane.org/2020-abstracts/developing-cat-hppr-critical-appraisal-tool-assess-
quality-systematic-rapid-and; 2020 [Accessed: 20.09.24].

3 Peters MD, Godfrey C, McInerney P, et al. Chapter 11: Scoping Reviews. In: Aromataris E, Munn Z, eds. JBI Manual for Evidence Synthesis 2020.

4 Tricco AC, Lillie E, Zarin W, et al. PRISMA Extension for Scoping Reviews (PRISMA-ScR): Checklist and Explanation. *Ann Intern Med* 2018;169(7):467–73. doi:10.7326/M18-0850 [published Online First: 4 September 2018].
