# Supplementary material for: Mental health among farmers in Germany: a scoping review
Source: Front Public Health. 2025 Dec 17;13:1701468. doi: 10.3389/fpubh.2025.1701468 (PMC12754006; doi:10.3389/fpubh.2025.1701468)
Supplement: Supplementary file 3 [file Data_Sheet_3.PDF]

Amendment – Study Protocol

Former title: Mental health among farmers in Germany: A rapid review

New title: Mental health among farmers in Germany: A scoping review

<u>Description of the amendment:</u>

The review protocol has been amended from a rapid review to a scoping review. This affects both the title as well as the methodology and guidelines used to conduct the planed review.

<u>Justification:</u>

The exploration of the literature revealed that the evidence base on the topic mental health among farmers in Germany is broader and more heterogeneous than anticipated, with widely varying study designs, populations. Further, the quality of the available studies vary enormously.

This variability limits the feasibility of conducting a rapid review aimed at synthesizing findings.

Adopting a scoping review methodology will enable us to comprehensively map the available literature regarding mental health among farmers in Germany and to identify research gaps, thereby ensuring the review remains methodologically appropriate and provides maximum relevance for future research and practice.
